# Ivy Poison

**Published:** 1912-09

**Authors:** 


Ivy Poison.—Robert T. Morris, of New York, recommends the following as treatment for "ivy poison:" As soon as the symptoms of itching and redness appear, wash the spots thoroughly with green soap and brush, followed with thorough washing with 98 per cent grain alcohol. He says that one treatment when used early cures the condition almost immediately. Dr. Morris writes this from personal experience after having been an annual sufferer for years. Having tried it on a number of cases besides his personal experience, he found the above treatment to afford the most rapid and perfect results obtained from any remedy he has ever tried.—*Journal A. M. A.*

*Medical Review of Reviews* (July 12) contains the following: As soon as signs of ivy poisoning appear wash very thoroughly with warm water and soap, then with bicarbonate of soda, 1 or 2 per cent solution, follow with several washings of 2 to 4 per cent permanganate of potash solution. Relief from itching is almost immediate and cure rapid.—*Lancet Clinic.*

---

At the San Francisco, Cal., meeting of the Federation of Women's Clubs the Committee on Sex Hygiene, through Mrs. M. W. Barry, of Sherman, Texas, Chairman, submitted the following as the sense of that committee:

"From the mass of reports and private correspondence the Committee on Sex Hygiene submits the following facts:

"1. That it is generally conceded by educators, physicians and social workers that there is urgent need for personal and sex hygiene instructions in our schools.

"2. That such instructions should be grounded on biology.

"3. That it should include more than mere physical facts.

"4. That it is dangerous to introduce into elementary and secondary schools until it is required in normal schools and teachers are carefully selected and prepared for the work.

"5. The instructions should be given to parents and gradually to the pupil.

"6. That popular prejudice against such instruction is rapidly disappearing.

"7. That such instruction is essential to eradicate the social evil and controlling disease."

